# What Makes for a Successful Digital Health Integrated Program of Work? Lessons Learnt and Recommendations From the Melbourne Children's Campus

**DOI:** 10.3389/fdgth.2021.661708

**Published:** 2021-05-24

**Authors:** Danah Hourani, Simone Darling, Eloise Cameron, James Dromey, Louise Crossley, Sanji Kanagalingam, Frank Muscara, Amanda Gwee, Grace Gell, Harriet Hiscock, Vicki Anderson

**Affiliations:** ^1^Murdoch Children's Research Institute, Melbourne, VIC, Australia; ^2^Department of Paediatrics, University of Melbourne, Parkville, VIC, Australia; ^3^Centre for Community Child Health, Murdoch Children's Research Institute, Melbourne, VIC, Australia; ^4^Curve Tomorrow, Melbourne, VIC, Australia; ^5^The Royal Children's Hospital, Melbourne, VIC, Australia; ^6^Health Services Research Unit, The Royal Children's Hospital, Melbourne, VIC, Australia

**Keywords:** digital health (eHealth), evidence-based & research methodology, pediatrics - children, health system - organization and administration, mobile application (app)

## Abstract

Embedding digital technologies in healthcare has the potential to streamline and personalize medical care. However, healthcare systems are often fragmented, and therefore achieving a truly integrated digital health program can be challenging. To promote a streamlined, evidence-based approach to implementing digital health solutions in a healthcare system, the Murdoch Children's Research Institute (MCRI) established the Digital Health Translation and Implementation Program (DHTI) bringing together clinicians, researchers and digital health experts. From the program commencement, frontline clinical innovators have collaborated with DHTI team members to develop and implement digital solutions to address pain-points in the healthcare system. Throughout this program, important lessons have been learnt relating to the development, evaluation and implementation of digital solutions in the healthcare system. This paper explores these lessons and makes recommendations for the successful implementation of digital health solutions in healthcare systems under five main categories: (1) design and usability, (2) stakeholder engagement and uptake, (3) project management and resourcing, (4) process and implementation, and (5) evaluation. Recommendations suggested here are designed to support future healthcare-based digital health programs to maximize the impact digital solutions can have on the healthcare system and patients.

## Introduction

Evidence-based digital technology has the potential to revolutionize healthcare, decreasing access barriers such as long wait-times and remote locations and increasing efficiencies during time-limited consultations (1, 2). The value of technology in healthcare has been highlighted during the COVID-19 pandemic, when evidence-based digital solutions, such as telehealth mobile or web applications (“apps”) and online portals, ensured the continuity of healthcare delivery (3–6). Consequently, the community could easily access evidence-based information and guidance on a range of pediatric conditions, clinical care continued remotely and research studies could collect crucial data during the pandemic (7–9). Yet the practicalities of implementation and sustainability of digital solutions in healthcare remain complex to navigate. To address these challenges, academic medical centers developed various programs (10–16). The Murdoch Children's Research Institute (MCRI) established the Digital Health Translation and Implementation program (DHTI) - an integrated program of digital health research and clinical and community application. The DHTI program, which commenced in 2017, aims to identify healthcare-related pain-points at the Royal Children's Hospital (RCH), and explore, develop and validate digital solutions [e.g., both standalone smartphone or web applications or those embedded into the Electronic Medical Records (EMR)] to solve these problems.

This paper details the structure, activities and essential elements of DHTI and aims to provide healthcare systems with a framework for successful development, implementation and sustainability of evidence-based digital health initiatives. Three such initiatives, developed and implemented by the DHTI program, are used to demonstrate the lessons learnt through establishing the program.

### DHTI Structure and Initial Consultation Framework

#### Team Structure

DHTI employs a multidisciplinary model including clinicians, researchers, and digital health experts and is supported by the Melbourne Children's Campus [“*the Campus”:* RCH, MCRI and University of Melbourne Department of Paediatrics (UMDP)], and regulatory, statistical and health economics experts. It leverages existing expertise within MCRI's digital health team and the in-house industry partnership with health technology company Curve Tomorrow (DHTI's working group), to identify, understand and address issues impacting delivery of clinical care (“pain-points”) identified by our clinical partner, The RCH (17). The program is managed by an executive team, with input from an International Advisory Group and a Program Advisory Committee (PAC), comprised of a multi-disciplinary group of Campus stakeholders who contribute to DHTI's identification of potential opportunities for implementation of digital health solutions.

An essential element of the DHTI program is the “clinical innovator” who has both clinical and digital health expertise and facilitates effective communication. They have protected time to identify and champion digital health opportunities, internally advocate for digital health solutions and work with the technical team to explore solution feasibility. Their input enables sustainable implementation within the healthcare system, embedding directly into clinical pathways, workflows and resources. In the late stages of development, clinical innovators facilitate knowledge transfer between clinical, research, innovation, implementation and evaluation working groups to assist with adopting digital solutions.

#### Campus Consultation

DHTI first consulted with Campus stakeholders across many RCH clinical programs about their pain-points. More than 40 interviews were conducted with RCH executives, heads of department, medical and allied health professionals, administration and support staff. Results were collated, and issues that met pre-determined selection criteria were retained. Criteria included: 1. The issue would be best solved using a digital solution, 2. Implementation was feasible given The RCH infrastructure, and 3. Solving the issue would align with the Campus strategic priorities (18). The selected issues were then considered by the DHTI executive and the PAC to determine the three most important issues, based on alignment with RCH pain-points, clinical, financial and operational impact, post-study implementation and sustainability.

### MCRI Innovation Lifecycle Stages

**Figure 1:**
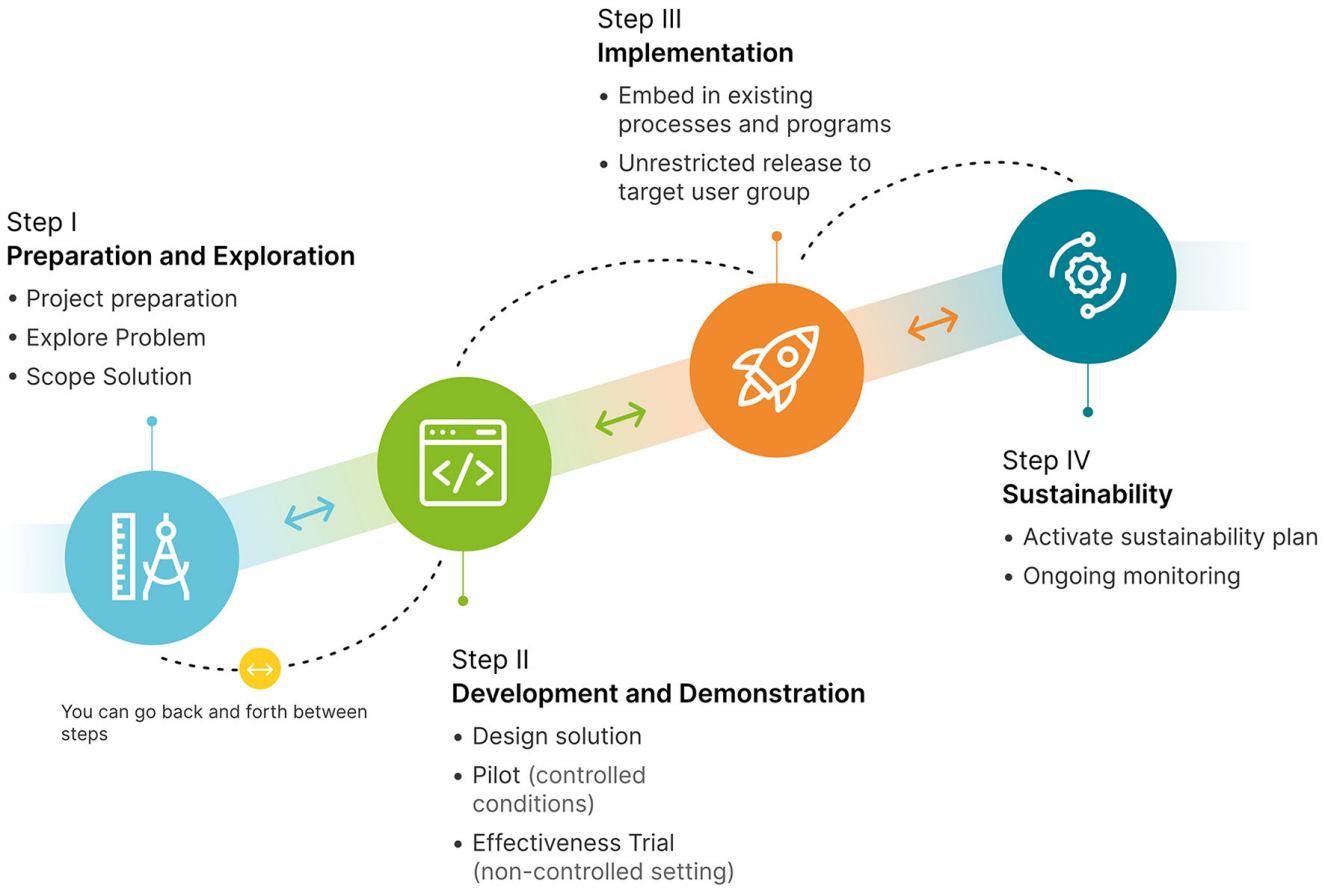
The MCRI innovation lifecycle stages.

For these initiatives, and subsequent digital solutions, DHTI developed a four step Innovation Lifecycle, to guide the development and implementation of solutions (Figure 1).

Step I, Preparation and Exploration, explores the problem and potential solutions in detail. Step II, Development and Demonstration, evaluates feasibility, usability, efficacy and effectiveness, often through pilot studies. Step III, Implementation, aims to embed the digital solution into routine practice. Step IV focuses on sustainability and requires a long-term vision and “business as usual” model—key to successful delivery of DHTI's solutions. While these steps are designed as sequential (i.e., problem exploration is completed before development), it can be necessary to return to earlier steps if problems are detected (i.e., new features need to be added at implementation, necessitating returning to exploration).

### DHTI Initiatives

DHTI focuses on developing “hospital-ready” digital solutions to address critical pain-points. Below we discuss three such initiatives that have successfully moved through the MCRI Lifecycle (Table 1). Each addresses a specific health problem and its corresponding digital solution, with clinical innovators working with clinicians, researchers, technology developers, behavior-change specialists and health care administration to take a product from step I (preparation and exploration) to step IV (sustainability), as per Figure 1. Lessons learnt throughout have been framed as recommendations in the following section.

**Table 1:** Description of DHTI initiatives.

**Pain-point**	**Initiative [Context] *Type of technology***	**Description**	**Key activities across Innovation Lifecycle**			
			**Step I – Preparation and exploration**	**Step II – Development and demonstration**	**Step III – Implementation**	**Step IV – Sustainability**
Available evidence-based concussion guidelines are not accessible to community sports coaches and families, leading to sub-optimal management and family uncertainty regarding children's return to normal activities	HeadCheck [Clinical, Industry, Community] *Smartphone application*	A smartphone app designed to assist caregivers to identify and act on concussion symptoms in children/adolescents, and most recently in adults. The app includes a concussion recognition component (sideline check and incident recording) and a recovery component [symptoms checking, recovery advice based on evidence-based clinical guidelines (19)]	Interviews with diverse range of stakeholders (major sporting codes, parents, coaches, first aiders, GPs, clinicians, teachers) to validate pain-point Landscape analysis of existing products that could solve pain-point Establish relationship with industry partner (AFL) Conduct user journey mapping Develop solution prototype Develop accurate budget and timeline for steps II–IV	Develop MVP Community pilot and market testing of usability Clinical pilot in emergency department to assess product efficacy and feasibility	Real world evidence: national launch Integration into multi-modal concussion intervention program Inclusion in mandatory training for coaches and first aid staff in community leagues	Leverage partnership with the AFL to launch HeadCheck Funding provided by AFL and venture capital group Freely available on iTunes and Google Play store Downloaded 59,000+ times and assisted in 4,500 head knocks (20)
Difficulty collecting accurate daily symptom data from healthcare workers in an international multicenter trial evaluating whether the Bacille Calmette-Guerin (BCG) vaccine may protect against COVID-19	MCRI Trial Symptom Tracker [Research] *Smartphone application*	This trial assesses the off-target efficacy of the BCG vaccine (for tuberculosis) in protecting against COVID-19 or reducing the severity of symptoms of COVID-19. The Trial Symptom Tracker is a smartphone app focused on participant engagement, and includes evidence-based behavior change techniques to retain participants	Review available evidence to establish which symptoms should be tracked as an indication of COVID-19 infection Consider user-friendliness for end-users (healthcare workers) and trial requirements for data collection Discuss behavior-change approaches to be integrated into app to ensure end-users seek COVID-19 testing when experiencing symptoms as per Department of Health guidelines	Evaluate participant uptake and data quality in a smaller group of participants to assess usability	Update app to also be available in Dutch and Spanish Ensure ethics approvals have been secured in each country where app is to be released Require participants download the app at randomization App compliance reviewed with participants at clinic visits	Plan to add additional data collection tools as COVID-19 environment changes, including new test methods and COVID-19 specific vaccines as they become available
Vancomycin (an antibiotic) is used for the treatment of serious bloodstream infections in young infants admitted to intensive care. Using the standard dosing guidelines, less than half (41%) achieve therapeutic levels (21)	Drug dosing calculator [Clinical] *Web-based application*	A web application that calculates the vancomycin dose for infants aged 0–90 days using a pharmacokinetic model. The calculator enables precise dosing based on specific patient characteristics and early drug monitoring to ensure that all babies achieve effective antibiotic concentrations for their infection.	Landscape analysis of existing dosing calculator (evaluating pros/cons) Ascertainment of perceived need/interest from end-users (medical, nursing, pharmacists) Feasibility assessment to integrate the calculator within the hospital electronic medical record Develop prototype Demonstration prototype and gather feedback from end-users Formulate project plan for step II	Evaluate barriers, enablers and calculator performance in a multicenter prospective cohort study in four neonatal intensive care units	Implementation in the hospital electronic medical record to streamline workflow and ensure safe prescribing practices	Plan for inclusion of the web app in neonatal dosing guidelines Leverage the web application platform to integrate other dosing calculators for children Explore opportunities for further integration within hospital medical records and online drug formularies

## Recommendations

While there were many learnings from the DHTI program, our working group determined that there were five main categories that captured the majority of recommendations for a general audience. Below we describe each of these recommendations within the context of the DHTI initiatives described in Table 1.

### Design and Usability

Design thinking is an established, iterative, solutions-based approach for ill-defined problems, involving input from multiple stakeholders to generate innovative solutions (22). Useful methodologies include mapping the patient journey and developing personas (23). The DHTI model uses Curve Tomorrow's “Design Way” (24), which is based on several published design thinking methodologies (25–28)., which ensures end users' experience is at the forefront at all stages of the Innovation Lifecycle.

The basic design thinking steps are: empathize, define, ideate, prototype, test and implement (29). For empathize, define and ideate, DHTI encourages spending enough time “exploring” potential solutions to avoid committing to a specific solution too early. Often the solution from this process will not be digital, but can be tested using a simple, low-cost prototype such as a pencil and paper solution. For example, a prototype of The Drug Dosing Calculator was first developed using Shiny application, allowing for rapid modifications to the product's core content before any costly development or EMR integration work commenced.

Testing usability is a key component of the development and demonstration step (step II). However, there are few digital health-specific published standards for usability, outside of guidelines in the field of health informatics (30). For example, the usability of HeadCheck was evaluated in several stages (20). Firstly, semi-structured interviews were run with emergency department (ED) consultants, nurse practitioners, sports medicine physicians, neurosurgeons, neuropsychologists, and physiotherapists to collect feedback on the acceptability, feasibility and utility of the app. Secondly, a community survey was conducted, identifying that 83.3% (*n* = 15) of parents agreed that the app helped them decide when to seek medical help for their child. Lastly, a sample of parents attending the ED with their child after their suspected concussion was surveyed, with 85.7% (*n* = 6) of parents reporting the app had increased their awareness of the importance of concussion recovery, knowledge of safe recovery, and timing for safe return to school/normal activities. This information was crucial for refining HeadCheck's design and functionality and demonstrates the importance of evaluating usability across a range of settings.

### Stakeholder Engagement and Uptake

Stakeholder engagement is increasingly encouraged to achieve sustainable research and clinical impact, yet target users are rarely included during conception or development phases (31). A principle critical to DHTI's success is involving stakeholders early in the Innovation Lifecycle, including end users, clinical champions and advocates who impact the decision-makers in the organization and/or the end users' behavior (32). In a pediatric context, involvement of stakeholders should be family-centered and include parents/guardians, teachers, siblings, family doctors and the children/adolescents themselves. The early involvement of stakeholders in problem and solution identification ensures a tailored solution design, facilitates engagement and creates a feeling of shared ownership (33). The ongoing involvement of stakeholders in the development and demonstration of the product ensures the product remains problem-focused, and the solution directly responds to target users' identified needs, maximizing the product's chance of successful long-term implementation and sustainability.

For community projects such as HeadCheck (20), industry partners [i.e., Australian Football League (AFL)] are critical to reach target audiences to allow research and clinical teams to canvass key insights as described in section Design and Usability. In this example, the AFL also advocated for the product, promoting it within their local and international networks and assisting with marketing and dissemination. The AFL were also aware of current and future industry-specific policies (e.g., concussion protocols), mandates and user pathways which are key in the Lifecycle of Innovation.

### Project Management and Resourcing

For each DHTI initiative, a core group was established, led by a project manager and supported by project-specific advisors. The responsibilities of the core group included appointing a “product owner”/“decision maker,” development of a program logic, ensuring clear documentation (product specifications) and transparency of deliverables with key stakeholders, formal agreements (license, collaboration), a project risk matrix and establishing clear, agreed roles and responsibilities to smoothen project execution. Project managers reported to the DHTI executive monthly to facilitate monitoring of progress and budgets.

DHTI initiatives were supported by a comprehensive budget to evaluate feasibility of initiatives prior to development, and guide approaches to potential partners and funding bodies. It is key to account for implementation and sustainability costs, facilitating product longevity beyond the development and demonstration (step II). In our experience, many of the costs associated with the development, implementation and sustainability of digital health solutions are poorly understood by clinicians and researchers. These include costs for technical support, app/website hosting, software upgrades, and regulatory approvals.

### Process and Implementation

Standardized processes and resources are important at both a program and project level. At the program level, DHTI uses a standardized intake and evaluation process for unearthing pain-points, and selecting projects to generate solutions. Since its inception, DHTI has assessed 17 digital projects as part of step I of the Innovation Lifecycle. Four (24%) products were excluded at phase 1 (Pre-screen), three (18%) products at phase 2 (Screen) and four (24%) at phase 3 (Due Diligence). Six products (35%) were selected to progress through to phases 4 and 5 (Recommendation, Approval and Commitment) before progressing to step II of the Innovation Lifecycle.

To assist with a standardized process at the project level, we developed the Digital Health Navigation System (Darling et al., unpublished manuscript), which is a “self-evaluation” tool for researchers and clinicians that consolidates resources and targeted information relevant to their product in an accessible, user-friendly format. It draws on published frameworks, models, standards, guidelines, and rating scales, relevant to implementing digital health technologies (e.g., developed by the World Health Organization) and emphasizes the importance of scientific rigor and evidence across all steps of the innovation process (34, 35). It aims to lift the standard of digital health, and creates accountability amongst all members involved in the innovation process.

While implementation comprises its own defined Step (Figure 1), it is critical to consider, from the outset of a project, that successful implementation of any health technology must occur within a complex multi-level system. Drawing on implementation science principles, the project team should consider not just the technology/product and the immediate user, but also the *inner* (immediate environment e.g., hospital department, school, GP clinic, home) and *outer* contexts (larger political, social and economic context) (36). Methods to explore and plan for this multi-level implementation include mapping existing organization systems and workflows involved in implementation (37), stakeholder engagement, usability measures, rigorous scientific evaluation (38–40) and planning for ongoing technical and clinical support post-launch, and budgeting appropriately. These aspects of planning can and should be revisited throughout the innovation lifecycle.

### Evaluation

DHTI has incorporated evaluation across all its Innovation Lifecycle stages, including technical and clinical validation, usability, and cost factors. An important component here is ensuring products meet standards set by relevant regulatory and governance bodies (e.g., Therapeutic Goods Administration, Food and Drug Administration, organization ethics committees), in each jurisdiction of users. For example, for the Trial Symptom Tracker to be released internationally 6 months after its initial release in Australia, a written submission was required by the Apple app store including evidence that the product complies with ethics regulations and was approved in all countries it was to be released in (Australia, UK, The Netherlands, and Spain). In a fast moving, live project where continuity of data collection was paramount, this posed a significant budget and time challenge.

Accepted ethical principles across clinical and health research, including respect for persons, beneficence, justice, and autonomy should be considered from the preparation phase to sustainability phase by all parties (developers, clinicians, researchers). Although issues of privacy are often evaluated, there are potentially implications for data quality and management, changes in patient-physician relationships and equity of access of healthcare services (41, 42). New ethical challenges continue to arise, and it is important to share knowledge and resources about identifying those challenges and steps to address them including the Connected and Open Research Ethics (CORE) initiative (43), Transparency for Trust (T4T) principles (44), and ethical navigation aids (45). Ethics committees may struggle to stay updated with ethical challenges emerging in the digital health space, so it is incumbent upon the digital health team to proactively find and utilize such resources.

Scientific evaluation is a challenging process in the digital health context, where rapid changes in technology are routine, and are incompatible with gold-standard research evaluation methodologies such as randomized control trials (RCTs) with typically elaborate prerequisites for study design suitability (28). In 2016, more than 259,000 health-related apps were available in app stores for smartphone devices and current numbers are likely much higher (46). However, few have undergone any scientific evaluation to determine effects on health outcomes, rather than use or user satisfaction, limiting their potential to impact clinical outcomes (47–49).

Optimally, evaluation is iterative and broad, based on needs and feedback, enabling the team to pivot to achieve the best solution. Digital health researchers need to identify novel, flexible approaches to evaluation and validation, and embrace real-world trials and evidence (50). DHTI's multicenter drug dosing calculator study, as mentioned in Table 1, exemplifies how to address this challenge through clearly defined research evaluation as an alternative methodology to an RCT design in digital health evaluation.

## Discussion

This article details the DHTI structure and activities, and proposed a framework to successfully develop, implement and sustain evidence-based digital health initiatives.

The lessons learnt from the Melbourne Children's Campus can be summarized as follows:

1. Maintain a problem-centered approach and do not neglect the initial exploration stage
2. Ensure true buy-in and representation from all relevant stakeholders by establishing a cross-disciplinary team of relevant consultants with expertise in software development, knowledge translation, behavior change, statistics, health economics, regulatory processes and healthcare
3. Budget accurately and consider “hidden” costs such as maintenance and support fees
4. Standardize processes and resources for a varied audience (clinicians, researchers) to develop, evaluate and implement solutions
5. Develop an evaluation framework that suits your local context, is flexible and applicable to a digital health content. Conduct thorough evaluation at every step of the process.

These important lessons should be interpreted within the context of several limitations. Firstly, while our team is confident of the utility of the MCRI Innovation Lifecycle and the DHNS, given they are based on published literature, multi-stakeholder expert input and extensive experience, neither resources have been empirically validated. Our team plans to evaluate the MCRI Innovation Lifecycle framework against other published innovation processes for usability and utility. Further, we plan to evaluate the DHNS using key success metrics e.g., reduction in adverse events, number of products successfully implemented in standard care, increased life expectancy of digital health products. Secondly, as DHTI is in its infancy, the guidance we provide around long-term sustainability (step IV) is limited. As the program matures, we will gather more learnings around sustainability of digital health solutions. Lastly, while our program emphasizes the inclusion of feedback from key stakeholders, we have not yet been able to incorporate feedback from young children and adolescents. This is a major future goal of the program and will be crucial to implementing successful products in a pediatric setting.

## Concluding Remarks

The novelty of digital health and its potential to rapidly impact and transform healthcare can raise concerns about credibility, reliability and success. A commitment by digital health programs such as DHTI to consistently, and rigorously evaluate at every step and incorporate feedback, will be essential to make stakeholders trusting and open to digital solutions.

DHTI successfully developed and implemented digital health solutions to solve real-world pain-points in the healthcare system, with important learnings captured throughout the process. While the DHTI program is by no means a perfect solution and will need to evolve with time, the key concepts described here are considered the most essential by the DHTI team for any digital health project. By prioritizing scientific rigor, ensuring standardized processes throughout the innovation lifecycle, and drawing on input from multi-stakeholders, programs such as DHTI play a crucial role in advocating for digital technologies' potential to transform how we deliver healthcare, optimizing care for our patients and broader community through rapid innovation and implementation.

As the first digital native generation approaches adulthood, the integration of digital technology into the lives of patients and the broader community will only increase. Healthcare systems need to embrace this digital revolution and ensure that the development of new processes and systems exist and the cultural shift has occurred to maximize readiness to adopt these solutions (51).

## Data Availability Statement

The original contributions presented in the study are included in the article/supplementary material, further inquiries can be directed to the corresponding author/s.

## Author Contributions

SD, DH, VA, and HH contributed the conception and brainstorming of the article. DH organized the structure of the article and wrote the manuscript. All authors contributed to the article and approved the submitted version.

## Conflict of Interest

The authors declare that the research was conducted in the absence of any commercial or financial relationships that could be construed as a potential conflict of interest.
